# Gender Equity Perceptions Among School-Going Adolescents: A Mixed-Methods Comparison Amongst Tribal and Non-Tribal Rural Areas of an Eastern State in India

**DOI:** 10.3389/fsoc.2021.772270

**Published:** 2022-02-03

**Authors:** Arista Lahiri, Sweety Suman Jha

**Affiliations:** Community Medicine, Dr. B. C. Roy Multi Speciality Medical Research Centre, Indian Institute of Technology, Kharagpur, India

**Keywords:** adolescent, gender-equity, gender-roles, gender-based violence, perception, tribal, mixed-methods research

## Abstract

**Introduction:** Gender equity is an important social determinant of population health. There are very few studies in the Indian context in this regard, and even less regarding the diversity in tribal-dominated and non-tribal regions. The current study was conducted to assess and compare the perceptions of adolescents going to selected schools of tribal-dominated and non-tribal rural areas in West Bengal, India, regarding gender equity. It also explored the teacher’s perceptions on gender equity in an attempt to contextualize the students’ perceptions.

**Methods:** A mixed-methods study was conducted with a convergent parallel design in two co-education schools, each from tribal-dominated and non-tribal rural areas. In the quantitative survey total of 828 adolescents aged 14—19 years participated. The qualitative component involved 26 in-depth interviews (IDIs) with the selected teachers.

**Results:** Overall, the respondents from tribal area had a better perception regarding the equitable privilege of independence among genders, equity in decision roles, and especially financial decision roles of women. Perceptions related to girls access to education were better among the boys from non-tribal area than their counterparts from tribal area. The non-tribal respondents had a more inadequate perception regarding women’s limited role. In general, the respondents perceived favorably against gender dominance. The teacher’s perceptions in the context were mostly concordant, with some exceptions, e.g., regarding dominance and violence-related issues, the teachers perceived differently, contrasting the better perceptions exhibited by the students.

**Conclusion:** The teachers’ perceptions showed strict reliance on the deep-rooted social norms that can be taken up for behavior change interventions. Better perceptions from the tribal areas are an opportunity to further enhance on gender equity. The boys’ perceptions can still be improved more in favor of gender equity.

## Introduction

The concept of gender equity, a critical social determinant of population health, has been gradually emerging as an area of prime importance to researchers (Phillips, 2005; Vlassoff, 2007; United Nations, 2010; World Health Organization, 2021). The term “gender” denotes the differences in social roles and their fulfillment. Heavily affected by prevalent social values and normative practices, gender-inequity is often cemented by gender discrimination in various functions despite no plausible explanation through biological differences in sexes (Braveman and Gruskin, 2003; Vlassoff, 2007). Gender inequity fueled by gender discrimination is globally considered to contribute to the growing burden of non-communicable and infectious diseases (Kim, 1990; Rathgeber and Vlassoff, 1993; Vlassoff and Manderson, 1998; Vlassoff, 2007; World Health Organization, 2019). The inequalities are perceived as deep-rooted and acculturated among backward, hard to reach, and marginalized areas, e.g., in some tribal-dominated regions (Vilms et al., 2017; Landry et al., 2019). This is often coupled with poor population health and poor healthcare access (Braveman and Gruskin, 2003).

Gender-equity as a whole has been a less studied topic in the Indian context concerning population health. The majority of the discussions are theoretical, with only a few studies to evaluate the empirical evidence regarding gender equity as a social determinant of health. These studies, however, revealed a situation nested in poverty, social adversity to women, and unjustifiably retrenched healthcare behavior with a hidden burden of morbidity, which is anything but far from equity (Brahmapurkar, 2017; Vilms et al., 2017; Landry et al., 2019). As per the recent data from National Family Health Survey 5 (NFHS-5), nearly half of the young married women surveyed were married before attaining the age of 18 years, one-fifth of the rural women had employment over past 1 year and property ownership, and nearly one-third of the ever-married women reported to have experienced spousal violence (Ministry of Health and Fa, 2021). Despite this challenging situation, literature on gender-equity are scarce in West Bengal.

The situation among marginalized or backward areas, e.g., tribal belt, requires an in-depth understanding, especially in contrast with non-marginalized areas. To understand the difference better and more precisely, search for possible prospective interventions to deal with this long-standing social problem; the school-going children’s, in particular, the late adolescent’s perspectives are cardinal, especially in the backdrop of their teacher’s perception of the same issue (Kolip and Schmidt, 1999; Jha et al., 2020). Informed by prior literatures, the comprehensive understanding of gender-equity among the adolescents was based on issues of independence, decision roles, access, competency in different roles, dominance and control, and idea regarding family sustainability (Dhal, 2018; Landry et al., 2019; Jha et al., 2020; Nanda; Vyas et al., 2020).

Grounded on the delicacy and complexity of the topic, an integrated approach through quantitative assessment of student’s perception along with an in-depth qualitative exploration of the teacher’s perspectives can effectively produce the desired yield utilizing the complementing strengths of both methodologies (Mägi, Biin, Trasberg, Kruus; Creswell, 2014). The current study thus, aimed to assess and compare the perceptions of adolescents going to selected schools of tribal and non-tribal rural areas in West Bengal, India, regarding gender equity. This study also explored the teacher’s perceptions on gender equity in an attempt to contextualize the student’s perceptions.

## Materials and Methods

### Study Design

A mixed-methods study was conducted with a convergent parallel design from August 2019 to January 2020. The data collection for the qualitative and quantitative parts was done simultaneously. The study was conducted in four rural higher secondary co-education schools in the southern part of West Bengal, India. Two schools were situated in the tribal-dominated rural fields of West Medinipur district, and the remaining two schools were located in rural areas of North 24 Parganas district, which was not a tribal-dominated area.

### Study Participants

Students of classes eight to twelve aged 14—19 years studying in the selected co-education rural schools of the study areas, who provided assent and whose parents provided consent for participation, were included in the cross-sectional survey. Students absent on the day of the survey were excluded from the study. The minimum required sample size for the quantitative part, calculated at 90% power and 5% precision based on the item-specific minimum score difference observed in the pilot study conducted beforehand was 348 adolescents from both the study areas. In the study areas based on total enrolment and proportional attendance two schools were selected from both tribal and non-tribal areas. In each selected school, from each included class one section was selected based on the principle of probability proportional to size. In the selected sections, all the students present were surveyed. Overall, from the four schools, 828 responses (397 from schools in the tribal area, and 431 from schools in the non-tribal area) were included for the survey part of the study. In the two schools from tribal area the average enrolment of the tribal students for the selected classes was 30% of the total. On the other hand, in the non-tribal area the enrolment of tribal students in the schools, on an average was around 11%.

The teachers of the schools who provided written informed consent and residing permanently in the adjoining tribal area (for tribal area schools) and non-tribal rural area (for non-tribal area schools) were included for the qualitative part of the study. However, teachers who have taught in the selected schools for a duration of less than one continuous year, or were not teaching the students of the selected classes were excluded. The respondents for the qualitative part were chosen conveniently based on the mentioned inclusion and exclusion criteria. The respondent teachers and students were not involved in the design, conduct, reporting, and dissemination plans of our research.

### Quantitative Measurement

The quantitative part of the study comprised an assessment of the adolescent’s perceptions and beliefs regarding gender equity with the help of a pre-designed pre-tested questionnaire—the questionnaire consisting of socio-demographic details of the respondents, and six selected domains related to gender equity, i.e., perceptions on “privilege of independence”, “decision role”, “access equitability”, “role competence”, “dominance and control”, and “family sustainability”. In the first phase of questionnaire development, the questions pertaining to gender equity were pooled together from other similar validated questionnaires (Men and Gender Equality Policy Project, 2011; Landry et al., 2019; Nanda). The pooled items were then assessed by an expert panel comprising of experts from the fields of Public Health (two), Sociology (one), and Psychology (two). A final brief version with relevant items was generated based on consensus among the experts by excluding redundant questions and incorporating items that were not in the pool but considered relevant by the experts (content validity ratio: 0.88). The questionnaire was then pre-tested on a sample of fifty adolescents of same age group, from separate rural co-education schools (one each from tribal and non-tribal areas). The final questionnaire consisted of six domains on gender-equity perceptions of the adolescents, with twenty questions assessed in three-point (i.e., agree, neither agree nor disagree and disagree) Likert-type scale.

Table 1 depicts the gender-equity questions as per domains. The reliability information for each domain is also mentioned. The questionnaire was translated to Bengali (the local language) by language experts and was back-translated to English by separate experts. The language was refined to achieve translational equivalence. The questionnaire (in English and local language) was administered for the survey among the participating students. The co-education schools were selected based on the number of students and average attendance from the list of the schools. In all the schools, there were multiple sections (two or three) in each class. Classes VIII to XII were chosen for the study. In each class, one section was randomly selected from each school. Classroom-based data collection was done for one section at a time, based on permission of the school authority, and availability of the students.

**TABLE 1:** Components and items in the gender equity questionnaire.

Statement	Responses		
	Agree	Partially agree	Disagree
**Privilege of independence** (Cronbach’s *α* = 0.774)			
1. Girls should be able to choose to work after marriage to earn their own money	719 (86.8)	62 (7.5)	47 (5.7)
2. Once a woman gets married, she belongs to her husband’s family	511 (61.7)	155 (18.7)	162 (19.6)
3. A woman should always obey her husband	490 (59.2)	216 (26.1)	122 (14.7)
**Decision roles** (Cronbach’s α = 0.693)			
4. Girls and boys should decide to do the same amount of housework	695 (83.9)	94 (11.4)	39 (4.7)
5. The husband should decide to buy the major household items	240 (29.0)	267 (32.2)	321 (38.8)
6. Girls should decide on their own about when to get married	531 (64.1)	103 (12.4)	194 (23.4)
7. Boys should decide on their own about when to get married	517 (62.4)	164 (19.8)	147 (17.8)
**Access equitability** (Cronbach’s α = 0.791)			
8. Boys should be fed before girls during meals	218 (26.3)	149 (18.0)	461 (55.7)
9. Boys should go to school over girls	118 (14.3)	142 (17.1)	568 (68.6)
10. Boys should get health services over girls	93 (11.2)	211 (25.5)	524 (63.3)
11. Since girls have to get married, they should not be sent for higher education	67 (8.1)	89 (10.7)	672 (81.2)
**Role competence** (Cronbach’s α = 0.725)			
12. Only boys can perform regular physical activity/work	242 (29.2)	282 (34.1)	304 (36.7)
13. Girls cannot do well in math or science	102 (12.3)	163 (19.7)	563 (68.0)
14. A woman can only take care of her home, kids and cook for her family	359 (43.4)	281 (33.9)	188 (22.7)
**Dominance and control** (Cronbach’s α = 0.688)			
15. There are times when a husband or a boy needs to beat his wife or girlfriend	83 (10.0)	88 (10.6)	657 (79.3)
16. A man should have the final word in his home and family matters	214 (25.8)	236 (28.5)	378 (45.7)
17. It is necessary to give dowry	78 (9.4)	126 (15.2)	624 (75.4)
**Family sustainability** (Cronbach’s α = 0.673)			
18. Only men should work outside the home to sustain the family	238 (28.7)	112 (13.5)	478 (57.7)
19. A woman should tolerate violence in order to keep her family together	200 (24.2)	225 (27.2)	403 (48.7)
20. A man using violence against his wife is a private matter that should not be discussed outside the couple	309 (37.3)	132 (15.9)	387 (46.7)

Values within the parentheses represent row percentages corresponding to the number of responses presented outside the parentheses.

### Qualitative Measurement

The qualitative component involved twenty-six in-depth interviews (IDIs) with the key informants among the selected teachers. In the non-tribal areas, fourteen teachers were interviewed, and from tribal areas, twelve teachers participated. The IDIs were conducted by the two researchers who had prior experience and training on qualitative research methods and lasted for 35–40 min in strict adherence to the interview guide developed beforehand. The IDI guide was prepared from the brainstorming sessions with three subject experts. It was further validated by five experts from the disciplines of Public Health (two), Sociology (one), and Psychology (two). The interview guide elicited issues such as “roles and responsibilities associated with each gender,” “Unequal privileges among the genders,” “practice of domination and compulsions,” and “access and freedom disparities between genders.” Audio recording and field notes were also taken during interviews. Data collection were continued till data saturation when no new information yielded from the interviews.

### Analysis

The survey responses were analyzed in STATA 14.2 (StataCorp, College Station, TX, United States ), and the qualitative data were analyzed through hand-code technique. The responses to each Likert-type item were scored from 1 to 3. The negative statements were reverse scored. Thus, establishing unidirectional scoring in the questionnaire so that a higher score for each item indicated a favorable perception towards gender equity. The domain-specific scores were calculated by averaging the linear combination of scores for the individual items that belonged to the domain in question, as shown in Table 1. A higher score in each domain indicated a favorable perception towards gender-equity in that domain and vice-versa. Domain specific scores and item scores were individually tested for normality by Shapiro-Wilk test and were found not normally distributed. Spearman correlations were calculated separately for tribal and non-tribal areas, explaining the correlation between the studied domains. Mann-Whitney U test was used to ascertain the statistical difference in different domains between tribal and non-tribal areas and between boys and girls in these areas separately.

For the IDIs, transcript generation and translation from local language to English were done within a day of the interview. Data collection and coding to find the critical segments were done simultaneously. Hand code technique was applied independently by two coders, and themes were generated. Transcripts were read multiple times initially to have a general understanding of the content. Codes were merged and summarized to form themes, and the themes prepared were further put into appropriate domains under the issues explored.

### Ethics

The current study was part of a larger study, which was approved by the Institutional Ethics Committee of Medical College and Hospital, Kolkata. The school’s authoritie’s permissions were taken before data collection with assent and consent obtained from the study participants.

## Results

### Study Participants and Their Characteristics

The respondent students were within the age group of 14–19 years with a mean (±standard deviation) age of 15.21 (±1.37) years. Participants belonging to scheduled tribes (ST), i.e., the tribal participants were more in the schools from tribal area (26.70%) compared to non-tribal area (8.12%), and this difference was statistically significant. Around 69.02% of students from tribal areas schools were female, while in the schools of non-tribal settings, and this was 60.09%. The gender differences of participants among the two study areas were statistically significant. The socio-demographic characteristics of the adolescent students are summarized in Table 2. Among the twenty-six teachers interviewed, the majority were Hindu (19), Male (15), and of general caste (14). Nine teachers belonged to the Scheduled Tribe category; six of them were from the tribal areas.

**TABLE 2:** Distribution of students according to socio-demographic characteristics and study area.

Socio-demographic characteristics		Study area				Total (*n* = 828)		*p*-value (χ^2^, df)
		Tribal (*n* = 397)		Non-tribal (*n* = 431)		*N*	*%*	
		*N*	*%*	*N*	*%*			
Class	VIII	127	32.0	158	36.7	285	34.4	0.059 (9.074, 4)
	IX	86	21.7	98	22.7	184	22.2	
	X	60	15.1	78	18.1	138	16.7	
	XI	72	18.1	51	11.8	123	14.9	
	XII	52	13.1	46	10.7	98	11.8	
Age group	≤15 years	252	63.5	295	68.4	547	66.1	0.142 (2.276, 1)
	>15 years	145	36.5	136	31.6	281	33.9	
Gender	Female	274	69.0	259	60.1	533	64.4	0.007 (7.177, 1)
	Male	123	31.0	172	39.9	295	35.6	
Religion	Hinduism	305	76.9	339	78.7	644	77.8	0.427 (1.701, 2)
	Islam	72	18.1	78	18.1	150	18.1	
	Others	20	5.0	14	3.2	34	4.1	
Caste	General	200	50.4	329	76.4	529	63.9	<0.001 (70.136, 3)
	OBC	49	12.3	32	7.4	81	9.8	
	SC	42	10.6	35	8.1	77	9.3	
	ST	106	26.7	35	8.1	141	17.0	

“n” represents the respective sample sizes. “N” implies the absolute frequency, and “%” indicates the column percentages for the respective categories. “χ^2^” indicates the chi-square statistic, and “df” indicates degrees of freedom. *p*-values are calculated based on the chi-square test/test of trend as applicable. OBC, other backward classes, SC, scheduled caste, and ST, scheduled tribes.

### Perceptions of the Students

Table 3 shows the scores of the adolescents in each of the items and also in each domain, comparing as per setting and gender. Overall, regarding perceptions on the privilege of independence and decision roles, respondents from tribal areas reported a higher score. However, there was a difference in perceptions on decision roles and family sustainability among the girls, which was statistically significant. The girls from tribal areas had a higher score in these domains than their counterparts from non-tribal areas. The respondents in general, and boys, in particular, had comparatively poorer scores (<2) in some items like, *“Once a woman gets married, she belongs to her husband’s family”*, *“A woman should always obey her husband” and “A woman can only take care of her home, kids and cook for her family”* irrespective of the area of residence, indicating poorer perception about these issues. Boys also had a lower perception score for items like “The husband should decide to buy the major household items” and “Boys should only perform regular physical activity/work than girls”. In general, the respondents from tribal areas had a better perception regarding gender equity compared to non-tribal respondents. But regarding the item “Girls should decide on their own about when to get married”, boys from non-tribal areas had a better perception compared to boys from tribal areas. The domain-wise perceptions of the students were correlated. The correlations were comparatively higher among the respondents from the tribal areas. The correlations are summarized in Supplementary Table S1.

**TABLE 3:** Domain-wise score comparison among respondents from tribal and non-tribal areas.

Statement	Comparison among boys				Comparison among girls				Overall comparison			
	Tribal (*n* = 123)	Non-tribal (*n* = 172)	Mann-whitney U*	*p*-value	Tribal (*n* = 274)	Non-tribal (*n* = 259)	Mann-whitney U*	*p*-value	Tribal (*n* = 397)	Non-tribal (*n* = 431)	Mann-whitney U*	*p*-value
**Privilege of independence**	**1.81 (0.30)**	**1.78 (0.31)**	**10121.00**	**0.466**	**2.11 (0.45)**	**2.06 (0.39)**	**33577.50**	**0.266**	**2.02 (0.43)**	**1.95 (0.39)**	**79029.5**	**0.045**
1. Girls should be able to choose to work after marriage to earn their own money	2.71 (0.64)	2.73 (0.57)	10512.00	0.896	2.87 (0.45)	2.85 (0.47)	34917.50	0.525	2.82 (0.52)	2.80 (0.52)	83578.5	0.328
2. Once a woman gets married, she belongs to her husband’s family	1.27 (0.57)	1.19 (0.51)	9863.00	0.113	1.80 (0.87)	1.75 (0.82)	34535.00	0.562	1.64 (0.83)	1.52 (0.76)	79934	0.059
3. A woman should always obey her husband	1.45 (0.72)	1.44 (0.76)	10284.50	0.611	1.66 (0.75)	1.58 (0.70)	33498.50	0.216	1.59 (0.74)	1.52 (0.73)	80746.5	0.111
**Decision roles**	**2.42 (0.39)**	**2.47 (0.36)**	**9774.50**	**0.252**	**2.49 (0.51)**	**2.36 (0.49)**	**29663.00**	**0.001**	**2.47 (0.48)**	**2.40 (0.45)**	**77094.5**	**0.013**
4. Girls and boys should decide to do the same amount of housework	2.85 (0.38)	2.91 (0.32)	9926.00	0.085	2.77 (0.54)	2.71 (0.60)	33875.50	0.188	2.80 (0.50)	2.79 (0.52)	85531.5	0.992
5. The husband should decide to buy the major household items	1.85 (0.71)	1.77 (0.64)	9946.00	0.335	2.31 (0.83)	2.20 (0.86)	33066.50	0.136	2.17 (0.82)	2.03 (0.81)	77347	0.011
6. Girls should decide on their own about when to get married	2.47 (0.72)	2.63 (0.64)	9353.00	0.041	2.42 (0.88)	2.22 (0.94)	31479.00	0.008	2.44 (0.83)	2.38 (0.85)	82779	0.342
7. Boys should decide on their own about when to get married	2.50 (0.69)	2.59 (0.63)	9923.00	0.284	2.48 (0.80)	2.30 (0.85)	31311.50	0.007	2.48 (0.77)	2.41 (0.78)	81042.5	0.128
**Access equitability**	**2.39 (0.51)**	**2.46 (0.49)**	**9661.00**	**0.197**	**2.59 (0.49)**	**2.54 (0.49)**	**33067.50**	**0.151**	**2.53 (0.51)**	**2.51 (0.49)**	**82637.5**	**0.380**
8. Boys should be fed before girls during meals	2.25 (0.88)	2.34 (0.87)	10023.50	0.386	2.32 (0.84)	2.25 (0.86)	33998.00	0.354	2.30 (0.85)	2.29 (0.86)	84979.5	0.852
9. Boys should go to school over girls	2.44 (0.75)	2.50 (0.78)	9940.00	0.230	2.62 (0.69)	2.54 (0.73)	33321.00	0.127	2.57 (0.71)	2.52 (0.75)	83528.5	0.472
10. Boys should get health services over girls	2.33 (0.78)	2.30 (0.79)	10412.00	0.801	2.65 (0.59)	2.62 (0.61)	34553.50	0.515	2.55 (0.67)	2.49 (0.71)	82004.5	0.227
11. Since girls have to get married, they should not be sent for higher education	2.54 (0.80)	2.71 (0.67)	9522.00	0.039	2.80 (0.49)	2.76 (0.52)	34364.00	0.342	2.72 (0.62)	2.74 (0.58)	84491	0.650
**Role competence**	**1.79 (0.46)**	**1.84 (0.47)**	**9829.50**	**0.289**	**2.35 (0.55)**	**2.29 (0.55)**	**33484.00**	**0.252**	**2.17 (0.58)**	**2.11 (0.56)**	**80541**	**0.139**
12. Only boys can perform regular physical activity/work	1.63 (0.63)	1.69 (0.66)	10130.00	0.492	2.29 (0.80)	2.31 (0.81)	35003.00	0.767	2.09 (0.81)	2.06 (0.81)	84071.5	0.647
13. Girls cannot do well in math or science	2.33 (0.81)	2.42 (0.79)	9902.00	0.291	2.69 (0.60)	2.62 (0.64)	33652.00	0.183	2.57 (0.69)	2.54 (0.71)	83464.5	0.460
14. A woman can only take care of her home, kids and cook for her family	1.41 (0.57)	1.42 (0.56)	10419.00	0.795	2.06 (0.82)	1.95 (0.80)	32808.00	0.110	1.86 (0.81)	1.74 (0.76)	78913	0.038
**Dominance and control**	**2.31 (0.61)**	**2.38 (0.59)**	**9857.50**	**0.307**	**2.64 (0.43)**	**2.57 (0.47)**	**32581.00**	**0.084**	**2.53 (0.52)**	**2.49 (0.53)**	**81298**	**0.197**
15. There are times when a husband or a boy needs to beat his wife or girlfriend	2.50 (0.82)	2.66 (0.70)	9672.50	0.097	2.78 (0.53)	2.71 (0.60)	33748.00	0.148	2.70 (0.65)	2.69 (0.64)	84915.5	0.793
16. A man should have the final word in his home and family matters	1.98 (0.85)	1.96 (0.86)	10410.00	0.805	2.38 (0.77)	2.27 (0.79)	32835.50	0.102	2.25 (0.81)	2.15 (0.83)	79474.5	0.057
17. It is necessary to give dowry	2.45 (0.80)	2.54 (0.77)	9907.00	0.258	2.77 (0.52)	2.73 (0.55)	34281.00	0.337	2.67 (0.64)	2.65 (0.65)	84731.5	0.751
**Family sustainability**	**2.05 (0.65)**	**2.11 (0.69)**	**9995.00**	**0.413**	**2.33 (0.60)**	**2.22 (0.59)**	**31592.50**	**0.026**	**2.24 (0.63)**	**2.18 (0.63)**	**80535**	**0.138**
18. Only men should work outside the home to sustain the family	1.96 (0.92)	1.99 (0.94)	10407.00	0.796	2.52 (0.78)	2.40 (0.84)	33006.50	0.094	2.35 (0.86)	2.24 (0.90)	80243.5	0.081
19. A woman should tolerate violence in order to keep her family together	2.26 (0.81)	2.36 (0.84)	9745.00	0.201	2.27 (0.81)	2.14 (0.81)	32145.50	0.044	2.27 (0.81)	2.23 (0.83)	83328.5	0.483
20. A man using violence against his wife is a private matter that should not be discussed outside the couple	1.94 (0.93)	2.00 (0.94)	10242.00	0.610	2.19 (0.90)	2.12 (0.88)	33869.00	0.321	2.12 (0.92)	2.07 (0.91)	83405.5	0.496

Values within the parentheses represent standard deviation corresponding to the mean value presented outside the parentheses. *p*-values were calculated by Mann-Whitney U test. *Denotes the test statistic observed in each test.

The numbers in bold represent the overall effect of these each domains.

### Qualitative Assessment

**FIGURE 1:**
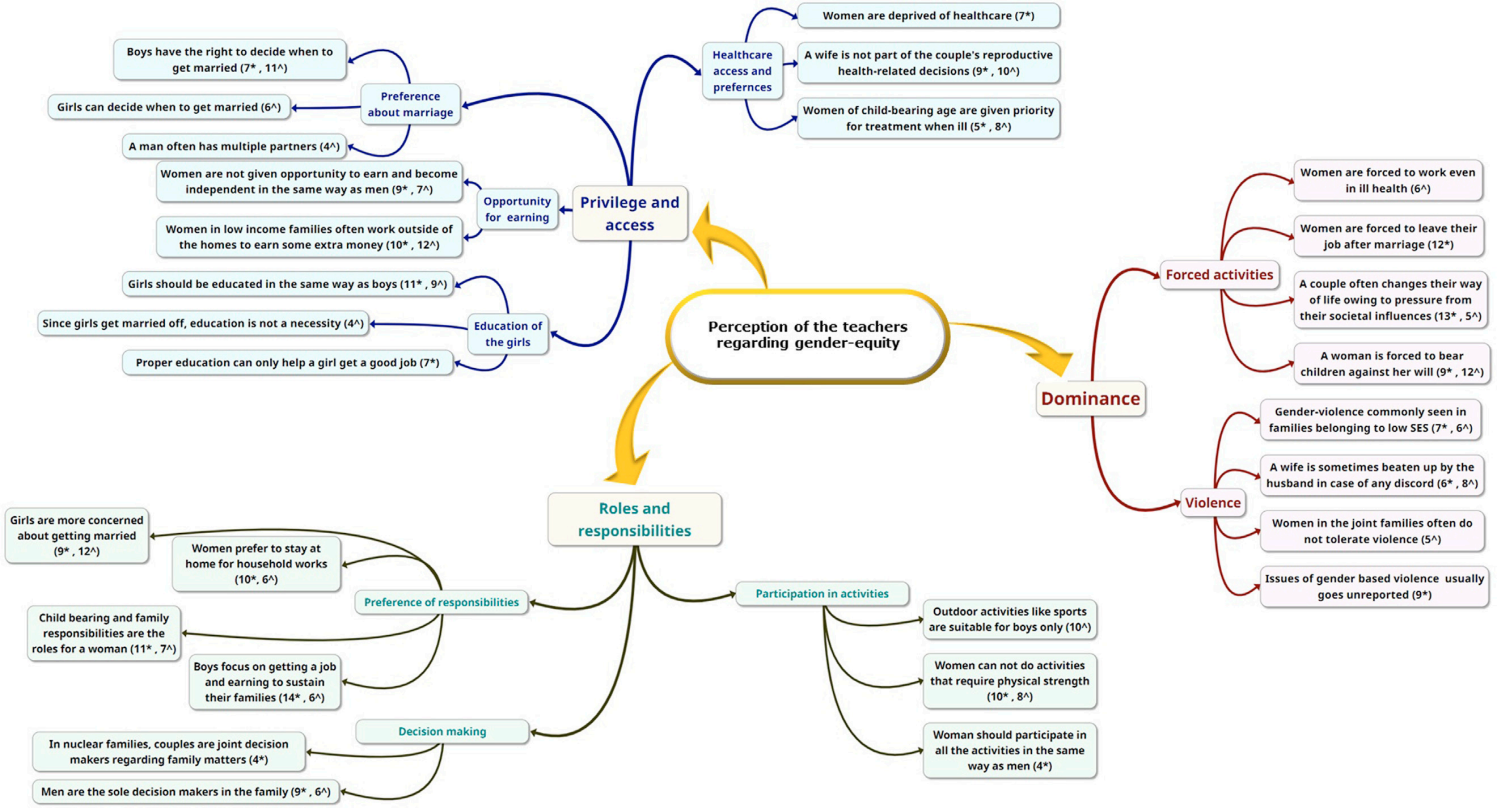
Themes from qualitative analysis. * respondents from non-tribal area, ^ respondents from tribal area.

The major themes that emerged from both tribal and non-tribal areas are depicted in Figure 1. The themes generated were alike for both tribal and non-tribal areas, except some themes exclusive to tribal or non-tribal areas and often discordant. The teachers perceived that *“boys have the right to decide when to get married”*, but particularly in tribal areas the teachers perceived the same about girls. In the tribal areas the teachers perceived that *“since girls get married off, education is not a necessity”*, “contrary to the teachers” opinion from non-tribal areas that *“proper education can only help a girl get a job”* and become independent. In this context, a female teacher of a school in a non-tribal area stated, *“Education is a must for a girl to get a job and to be able to live independently.”*

The teachers from non-tribal areas also perceived that *“women are forced to leave their job after marriage”*. Deprivation of women was perceived in both settings. In non-tribal areas teachers perceived that *“women are deprived of healthcare”,* and in tribal areas, the teachers felt that *“women are forced to work even in ill health*. Interestingly, women of child-bearing age were given priority for treatment access in both areas. A male teacher in tribal areas perceived”, *“In small families, women are left with no choice but to work and take up the household activities, even if they are ill”*. From non-tribal areas, a senior male teacher commented, *“Women are not taken to hospitals or dispensaries; healthcare of a girl or an elderly lady is not important to the family. Only a woman who can and will bear children is given some priority to provide treatment when needed.”*

It was explored that in some tribal families, women do not tolerate violence, but on the contrary, in non-tribal areas, gender violence often remained hidden, and unreported. A female teacher in tribal areas responded, *“Here the women work hard day and night to take care of the family; they do not tolerate the beatings or any abusive treatment from the husband”*. While men were perceived to be the sole decision-maker in the study areas, in non-tribal areas, teachers opined that *“in nuclear families, couples are joint decision-makers regarding family matters.”*

### Integration of the Findings

**FIGURE 2:**
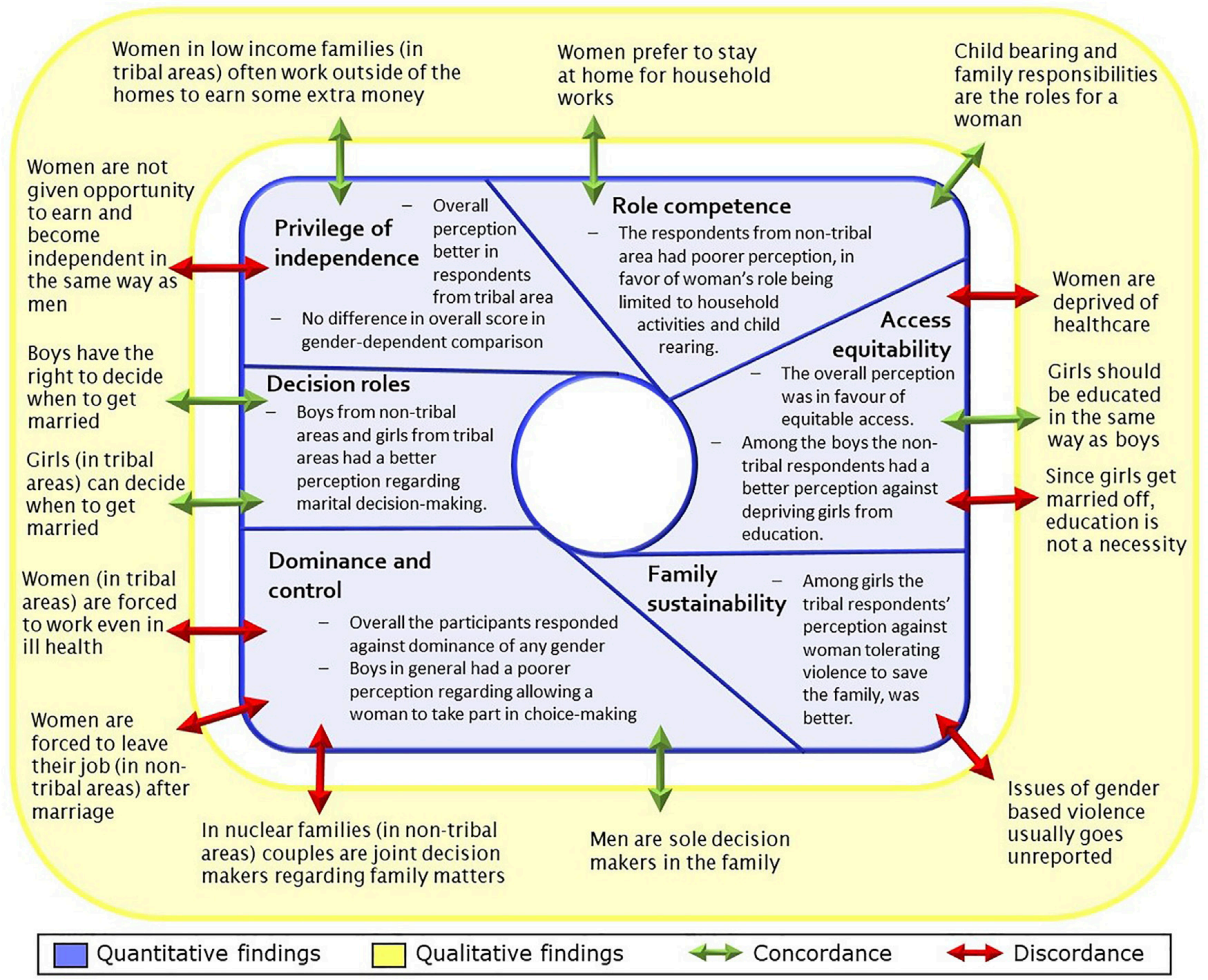
Integration of quantitative findings with qualitative findings.

Figure 2 represents the integration of qualitative and quantitative findings. The findings were concordant primarily. Regarding access equitability, qualitative discordance was noted for deprivation of women of healthcare and education. Again, in the domain of dominance and control, though the students had a perception in favor of gender equity, the teachers explained how social norms are dominant and deprive women of their rights.

## Discussion

### Key Findings

This mixed-methods study described the perceptions relating to gender-equity among the adolescents in tribal and non-tribal rural areas, contextualizing through the perceptions of their teachers. Access equitability was found to be correlated with role competence, and dominance and control domains. The correlation was stronger among respondents from tribal areas, which was consistent with the study’s overall findings. Overall, the respondents from tribal areas had a better perception regarding the equitable privilege of independence among genders. A similar difference was observed for the perception of equity in decision roles, especially among the respondent girls. In general, regarding the financial decision roles of women, respondents from tribal areas had a comparatively better perception. Regarding marriage decisions, a similar trend was observed, with the exception of respondent boys from tribal areas who had a relatively inadequate perception regarding a girl’s marriage decisions. Perceptions related to girls access to education were better among the boys from non-tribal areas compared to their counterparts from tribal areas. Overall, the non-tribal respondents had more inadequate perception in favor of women’s limited role. The mean score regarding role competence among genders was comparatively lower overall and across genders. Overall, the respondents perceived favorably against gender dominance. Among girls, the perception regarding the equitable role of genders in sustaining a family was better in tribal areas. Among the girls, the tribal respondent’s perception against women tolerating violence to save the family was better. The teacher’s perceptions were used to contextualize the students’ responses. They were mostly concordant in nature, with some exceptions, e.g., regarding dominance and violence-related issues, the teachers perceived differently contrasting the better perceptions exhibited by the students.

### What is Already Known and What This Study Adds

In a previous study among adolescents from a tribal-dominated rural area, Jha et al. demonstrated that girls had a better attitude regarding gender equity compared to boys (Jha et al., 2020). Landry et al., in their cross-sectional study in Northern India, in a similar way found out that boys were less likely to develop a gender-equitable attitude compared to girls (Landry et al., 2019). However, in the current study, respondent boys had an overall favorable perception of gender-equity in almost all domains. The boys’ perceptions were comparatively poorer while discussing male dominance of different roles. Interestingly, among girls, the overall perception related to family sustainability was better in tribal areas. But this was contrasted by the teacher’s opinion of frequent under-reporting or no-reporting of gender-violence issues. The complex contradiction evidenced can be explained in terms of the dynamics of socio-economic disadvantage-driven equity in social sustainability and gender roles and normative male dominance in rural areas in general.

Studies have indicated that progressive influence on social institutions focusing on sharing responsibilities among genders and men assuming more non-traditional roles is necessary to overcome gender discrimination (King et al., 2018; England et al., 2020). Bhasin V. discussed the prevalent gender disparities in India and outlined its variation in the tribal communities and the perceived importance of a tribal woman’s role extending beyond the segregation of economic outputs (Bhasin, 2007). The current findings support this discourse. The adolescent’s perceptions were more in favor of girl’s versatile and independent role in the backdrop of their teacher’s similar perception of women from tribal areas going out of their homes to work and earn extra money. But on the other hand, teachers from both the study areas felt that girls are not usually given proper opportunities or the motivation to move forward. This was in line with the findings of Keynejad et al. (2018). The student’s perceptions about equal access to education for girls were contradicted by the teachers of tribal areas, who perceived education as a mere auxiliary compared to the principal role of marriage. The normative judgment of the preceding social institutions justifies this contradiction (Bhasin, 2007).

In rural areas of the eastern part of the country, healthcare access has been perceived as gender-discriminant in healthcare expenditure or access to facility-based curative or preventive care (Saikia and MoradhvajBora, 2016; Vilms et al., 2017; Jha et al., 2020). The teacher’s perceptions explored were, however, in accordance with these findings. The current qualitative results highlight that women’s marital and reproductive role is considered to be of utmost importance, which is not only a social norm, but also prioritized. Though in a discordant manner, the students perceived mostly in favor of gender-neutral access to services and associated freedom of choice. The tribal areas students had a comparatively better attitude. But regarding girls own choice/decision of marriage, the boys from non-tribal areas had a comparatively better perception. This may indicate an underlining patriarchal system dominating the tribal or marginalized areas, explaining the tribal girls better perception of women’s roles, which the elderly members often contradict. The findings are similar to what Walia et al., in their study conducted in a rural area of North India, concluded about men’s participation and dominance in reproductive decision making (Walia et al., 2021). The social norms and role-stereotyping of genders have been documented similarly by researchers in India and abroad, emphasizing tribal and disadvantaged populations (Khanna et al., 2018; Willan et al., 2020).

Keynejad et al. reported their young respondents’ perception of gender-based violence being a major cause for concern (Keynejad et al., 2018). However, the students’ perceptions of dominance in the current study were in stark contrast to what the researchers have demonstrated previously. But the teacher’s responses were divergent and directed towards violence and domination by the male gender. The socio-economic perspective of social dynamics can explain this in a tribal context similar to what Dhal S. explored (Dhal, 2018). The teacher’s perceived that often women in tribal areas are forced to forego their health priorities, a situation similar to what Sethuraman et al. and Jha et al. explored among rural tribal communities (Sethuraman et al., 2006; Jha et al., 2020).

### Strengths and Limitations

In the current research topic, quantitative and qualitative analyses complemented each other with the advantage of strength in each methodology. As a result, the convergence and divergence in findings provided an understanding. A clear idea of prevalent perceptions within the education system was obtained by including both students and teachers. The current study was probably the first study to explore gender equity perception among adolescents in different rural settings and compare them among tribal and non-tribal areas. The method of conducting the survey and arrangement of the classroom setting attempted to minimize dilution and social desirability. Still, these might have had an impact, resulting in non-interrelated response sets to items in some domains showing lower value of Cronbach’s alpha. However, despite the robustness in the mixed-methods design and the study’s conduct, the chance of social desirability bias in individual responses cannot be undermined. The results show the situation of the selected areas only. The variation in responses might have occurred due to variation in cognitive levels of the students and age. But these were out of scope for the current study and remained unanswered, paving for further research.

## Conclusion

The past half a century has been termed as an era of “gender revolution”. Yet, even in the most pragmatic societies, the strive for equity has stalled a bit. The current study is among only a few studies conducted in India that perform a critical investigation in the adolescent students’ perception of the multi-dimensional concept of gender-equity, an essential determinant of health. The comparison of responses among tribal and non-tribal areas presents a rural epiphany of gender-equitable perceptions of adolescents. Their teacher’s perception, however, highlights the deep-rooted social norms which are functional areas for behavior change interventions. It is observed that health reforms and new models of governance usually do not take into account the gender perspective, which often acts endogenously in the models (Kuhlmann et al., 2015). The current evidence empirically dictates in favor of accounting for gender discrimination while devising newer interventions. Researchers have established gender as a complex determinant of health. Considering the complications posed by the COVID-19 pandemic, Connor et al. have predicted an expanding gender difference in health risks, which can push society to the brink of extreme vulnerability (Connor et al., 2020). Therefore, taking actions to improve gender equity is a way to address women’s right to health, which can improve society’s overall health through the effective utilization of health resources. Better perceptions from the tribal areas are an opportunity to further enhance on gender-equity. However, boys having a comparatively unfavorable perception of equity for woman’s participation in decision making, freedom of working outside, and roles other than child-rearing and family care, etc., are areas where policy-makers need to focus, as this can only intensify gender discrimination in a future society. Formulation of gender-sensitive socially deliverable policies is thus advocated.

## Data Availability

The original contributions presented in the study are included in the article/Supplementary Material, further inquiries can be directed to the corresponding author.

## References

[B1] Bhasin V. (2007). Status of Tribal Women in India. Stud. Home Community Sci. 1 (1), 1–16. 10.1080/09737189.2007.11885234

[B2] Brahmapurkar K. P. (2017). Gender equality in India Hit by Illiteracy, Child Marriages and Violence: a Hurdle for Sustainable Development, Internet. Pan Afr. Med. J. 28, 178. cited 2019 Aug 6. 10.11604/pamj.2017.28.178.13993 29541324 PMC5847257

[B3] Braveman P. Gruskin S. (2003). Poverty, Equity, Human Rights and Health. Bull. World Health Organ. 81 (7), 539–545. 12973647 PMC2572503

[B4] Connor J. Madhavan S. Mokashi M. Amanuel H. Johnson N. R. Pace L. E. (2020). Health Risks and Outcomes that Disproportionately Affect Women during the Covid-19 Pandemic: A Review. Soc. Sci. Med. 266, 113364. 10.1016/j.socscimed.2020.113364 32950924 PMC7487147

[B5] Creswell J. W. (2014). Research Design: Qualitative, Quantitative, and Mixed Methods Approaches. 4th ed. Thousand Oaks: SAGE Publications, 273.

[B6] Dhal S. (2018). Situating Tribal Women in Gender Discourse: A Study of the Socio-Economic Roots of Gender Violence in Odisha. Indian J. Public Adm. 64 (1), 87–102. 10.1177/0019556117735459

[B7] England P. Levine A. Mishel E. (2020). Progress toward Gender equality in the United States Has Slowed or Stalled. Proc. Natl. Acad. Sci. U S A. 117 (13), 6990–6997. 10.1073/pnas.1918891117 32229559 PMC7132302

[B9] Jha S. S. Dasgupta A. Paul B. Ghosh P. Biswas A. (2020). Attitude and Perception of Gender Equity Among Students and Teachers of a Rural School in West Bengal: A Mixed-Method Approach. J. Educ. Health Promot. 9 (1), 330. 10.4103/jehp.jehp_597_20 33575366 PMC7871973

[B10] Keynejad R. C. Mekonnen F. D. Qabile A. Handuleh J. I. M. Dahir M. A. Haji Rabi M. M. (2018). Gender equality in the Global Health Workplace: Learning from a Somaliland-UK Paired Institutional Partnership. BMJ Glob. Health 3 (6), e001073. 10.1136/bmjgh-2018-001073 PMC6304104 30613426

[B11] Khanna T. Chandra M. Singh A. Mehra S. (2018). Why Ethnicity and Gender Matters for Fertility Intention Among Married Young People: a Baseline Evaluation from a Gender Transformative Intervention in Rural India. Reprod. Health 15 (1), 63. 10.1186/s12978-018-0500-0 29653571 PMC5899360

[B12] Kim J. S. (1990). Sex Role Effects on Female Response to Illness. Korea J. Popul. Dev. 19 (2), 135–155. 12343388

[B13] King T. L. Kavanagh A. Scovelle A. J. Milner A. (2018). Associations between Gender equality and Health: a Systematic Review. Health Promot. Int. 35 (1), 27–41. 10.1093/heapro/day093check 31916577

[B14] Kolip P. Schmidt B. (1999) Gender and health in adolescence / by Petra Kolip and Bettina Schmidt. Copenhagen: WHO Regional Office for Europe, 38. Available from: https://apps.who.int/iris/handle/10665/108178

[B15] Kuhlmann E. Annandale E. (2015). “Gender and Healthcare Policy,”. Internet in The Palgrave International Handbook of Healthcare Policy and Governance. Editors Kuhlmann E Blank RH Bourgeault IL Wendt C (London: Palgrave Macmillan UK), 578–596. cited 2019 Aug 6, Available from:. 10.1057/9781137384935_35

[B16] Landry M. Vyas A. Malhotra G. Nagaraj N. (2019). Adolescents’ Development of Gender Equity Attitudes in India. Int. J. Adolescence Youth 25 (1), 94–103. 10.1080/02673843.2019.1590852

[B17] Mägi E. Biin H. Trasberg K. Kruus K. Gender Awareness and Attitudes toward Gender equality Among Students Participating in Teacher Training, 4.

[B18] Men and Gender Equality Policy Project. International Men and Gender Equality Survey (IMAGES) [Internet]. 2011 [cited 2021 Dec 4]. Available from: https://www.icrw.org/wp-content/uploads/2016/10/International-Men-and-Gender-Equality-Survey-IMAGES.pdf

[B19] Ministry of Health and Family Welfare (2021). National Family Health Survey - 5 (2019 - 2020)| State Fact Sheet: West Bengal. [Internet]. New Delhi: International Institute of Population Sciences. Available from: http://rchiips.org/nfhs/NFHS-5_FCTS/West_Bengal.pdf cited 2021 Dec 4.

[B20] Nanda G. Compendium of Gender Scales. Washington, DC: FHI 360/C-Change. Available from: http://gender.careinternationalwikis.org/_media/c-change_gender_scales_compendium.pdf.

[B21] Phillips S. P. (2005). Defining and Measuring Gender: A Social Determinant of Health Whose Time Has Come, Internet. Int. J. Equity Health 4 (1), 11. cited 2019 Oct 27. 10.1186/1475-9276-4-11 16014164 PMC1180842

[B22] Rathgeber E. M. Vlassoff C. (1993). Gender and Tropical Diseases: a New Research Focus. Soc. Sci. Med. 37 (4), 513–520. 10.1016/0277-9536(93)90286-d 8211263

[B23] Saikia N. Moradhvaj Bora J. K. (2016). Gender Difference in Health-Care Expenditure: Evidence from India Human Development Survey. PLOS ONE 11 (7), e0158332. 10.1371/journal.pone.0158332 27391322 PMC4938214

[B24] Sethuraman K. Lansdown R. Sullivan K. (2006). Women’s Empowerment and Domestic Violence: The Role of Sociocultural Determinants in Maternal and Child Undernutrition in Tribal and Rural Communities in South India. Food Nutr. Bull. 27 (2), 128–143. 10.1177/156482650602700204 16786979

[B25] United Nations (2010). Global Strategy for Women’s and Children’s Health. Internet. cited 2019 Aug 6, Available from: https://www.who.int/pmnch/topics/maternal/20100914_gswch_en.pdf?ua=1 .

[B26] Vilms R. J. McDougal L. Atmavilas Y. Hay K. Triplett D. P. Silverman J. (2017). Gender Inequities in Curative and Preventive Health Care Use Among Infants in Bihar, India, Internet. J. Glob. Health 7 (2). 020402, cited 2019 Aug 6. 10.7189/jogh.07.020402 28959437 PMC5592115

[B27] Vlassoff C. (2007). Gender Differences in Determinants and Consequences of Health and Illness. J. Health Popul. Nutr. 25 (1), 47–61. 17615903 PMC3013263

[B28] Vlassoff C. Manderson L. (1998). Incorporating Gender in the Anthropology of Infectious Diseases. Trop. Med. Int. Health 3(12):1011–1019. 10.1111/j.1365-3156.1998.tb00001.x 9892287

[B29] Vyas A. N. Malhotra G. Nagaraj N. C. Landry M. (2020). Gender Attitudes in Adolescence: Evaluating the *Girl Rising* Gender-Sensitization Program in India. Int. J. Adolescence Youth 25 (1), 126–139. 10.1080/02673843.2019.1598450

[B30] Walia M. Mittal A. Kumar D. (2021). Male Participation in Reproductive Health Care of Women and Factors Associated with Interpersonal Relationship: A Cross-Sectional Study in a Rural Community of Ambala District in Haryana. Indian J. Public Health 65 (2), 178–184. 10.4103/ijph.IJPH_262_20 34135188

[B31] Willan S. Gibbs A. Petersen I. Jewkes R. (2020). Exploring Young Women’s Reproductive Decision-Making, agency and Social Norms in South African Informal Settlements. PLOS ONE 15 (4), e0231181. 10.1371/journal.pone.0231181 32348303 PMC7190118

[B32] World Health Organization. WHO | Women and Gender Equity [Internet].WHO. [cited 2021 December 12]. Available from: https://www.who.int/health-topics/gender#tab=tab_1

