# Supplementary material for: Gender Equity Perceptions Among School-Going Adolescents: A Mixed-Methods Comparison Amongst Tribal and Non-Tribal Rural Areas of an Eastern State in India
Source: Front Sociol. 2022 Feb 3;6:772270. doi: 10.3389/fsoc.2021.772270 (PMC8853706; doi:10.3389/fsoc.2021.772270)
Supplement: Supplementary file 1 [file Table1.DOCX]

**Gender Equity Perceptions among School-going Adolescents: A Mixed-Methods Comparison amongst Tribal and Non-tribal Areas of an Eastern State in India**

Supplementary Material

# Supplementary Table

**Table S1. Correlation of the perceptions on domains of gender equity among the respondents from tribal and non-tribal areas.**

|  | Privilege of independence | Decision roles | Access equitability | Role competence | Dominance and control | Family sustainability |
| --- | --- | --- | --- | --- | --- | --- |
| Privilege of independence |  | 0.289  (0.000) ^b^ | 0.343  (0.000) ^b^ | 0.493  (0.000) ^b^ | 0.297  (0.000) ^b^ | 0.457  (0.000) ^b^ |
| Decision roles | 0.044  (0.365) ^a^ |  | 0.190  (0.000) ^b^ | 0.202  (0.000) ^b^ | 0.251  (0.000) ^b^ | 0.353  (0.000) ^b^ |
| Access equitability | 0.248  (0.000) ^a^ | 0.100  (0.038) ^a^ |  | 0.646  (0.000) ^b^ | 0.627  (0.000) ^b^ | 0.606  (0.000) ^b^ |
| Role competence | 0.500  (0.000) ^a^ | 0.067  (0.167) ^a^ | 0.582  (0.000) ^a^ |  | 0.491  (0.579) ^b^ | 0.579  (0.000) ^b^ |
| Dominance and control | 0.231  (0.000) ^a^ | 0.162  (0.001) ^a^ | 0.561  (0.000) ^a^ | 0.438  (0.781) ^a^ |  | 0.564  (0.000) ^b^ |
| Family sustainability | 0.348  (0.000) ^a^ | 0.219  (0.000) ^a^ | 0.598  (0.000) ^a^ | 0.519  (0.000) ^a^ | 0.579  (0.000) ^a^ |  |

The values in the cells represent spearman coefficients, and the values in parentheses represent their respective P-values.

**^a^:** correlation between domains among the respondents from the non-tribal area, **^b^:** correlation between domains among the respondents from the tribal area
